# The Neural Markers of Self-Caught and Probe-Caught Mind Wandering: An ERP Study

**DOI:** 10.3390/brainsci11101329

**Published:** 2021-10-08

**Authors:** Yong Liu, Jia Zhao, Xinqi Zhou, Xiaolin Liu, Hong Chen, Hong Yuan

**Affiliations:** 1 Key Laboratory of Cognition and Personality (Ministry of Education), Southwest University, Chongqing 400715, China; liuy0768@swu.edu.cn (Y.L.); jiazhao@swu.edu.cn (J.Z.); linzi306093@email.swu.edu.cn (X.L.); chenhg@swu.edu.cn (H.C.); 2 School of Psychology, Southwest University, Chongqing 400715, China; 3 School of Life Science and Technology, University of Electronic Science and Technology of China, Chengdu 611731, China; 201711090103@std.uestc.edu.cn; 4 Chongqing Institute of Foreign Studies, Chongqing 400715, China

**Keywords:** self-caught mind wandering, probe-caught mind wandering, neural markers, mSART

## Abstract

Mind-wandering (MW) is a common phenomenon, defined as task-unrelated thoughts. This study is based on event-related potentials (ERPs), using modified sustained attention to response task (modified SART, mSART) to discuss the neural patterns of different types of MW. In the current study, we defined the MW realized by participants as self-caught MW, and the MW measurement acquired by probes as probe-caught MW. The behavioral results showed that the reaction times (RTs) during self-caught MW were greater than those during non-self-caught MW. The ERP results showed that during self-caught MW, the mean amplitudes of N1 decreased significantly, indicating that the participants’ attention had deviated from the current task. The increase in the mean amplitudes of P2 during self-caught MW indicated lower vigilance. We also found that the mean amplitudes of N300 reduced during self-caught MW, which indicated that cognitive control or monitoring might be affected by self-caught MW. The average amplitudes of P300 were significantly lower during probe-caught MW than during on-task, indicating the impact on high-level cognitive processing. In addition, the amplitudes of N1, P2, and N300 in anterior regions were greater than those in posterior regions. P300 amplitudes during probe-caught MW in the right hemisphere were greater than those of the left hemisphere. In summary, our research results demonstrated that alertness and cognitive processing decreased during both self-caught MW and probe-caught MW. ERPs were statistically different under the conditions of self-caught MW and probe-caught MW. The current study provided new insights into the relationship between MW and neural markers. It was the first study exploring the ERP correlates between self-caught MW and probe-caught MW based on mSART.

## 1. Introduction

Mind-wandering (MW) is a common human experience. MW episodes are related to the emergence of task-unrelated thoughts and affects that divert attention from the task at hand [1,2]. For example, we plan to read a book. Usually, after reading for a while, we may recall a beautiful autumn day, with golden ginkgo leaves covering the forest, warm sunlight filtered through the treetops or something. After some time, we suddenly realize that our minds are wandering, drawing our attention back to the book. In other words, when we are MW, we are thinking about things other than the task at hand. Sometimes we are actively aware of these experiences, but sometimes we are passively aware when prompted. However, MW involves a “smooth transition” from considering the current task to thinking about other things [3].

The brain’s responses are blunted because attention is directed “inwardly” when we are MW [4]. MW has harmful effects on the performance of many cognitive tasks [5,6]. When we are MW, our alertness and vigilance become lower [7]. Therefore, studying the physiological mechanisms underlying MW is crucial to understand how to improve attention.

Previous studies on the nature of meta-awareness provide evidence that individuals routinely (at least temporarily) fail to notice that they are MW [8,9,10]. Schooler and colleagues theorized that MW reflects the cyclical activity of two core processes: the capacity to disengage attention from perception and the ability to pay attention to the current contents of consciousness. The former is known as perceptual decoupling, while the latter is called meta-awareness. Meta-awareness is defined as one’s explicit knowledge of the current thought contents. It is an intermittent process in which individuals periodically notice the current contents of their minds. They believed that these two processes were particularly useful in revealing the extent to which individuals were only intermittently aware of MW. In addition, MW was an indicator of these two attention fluctuations, and meta-awareness may directly regulate MW [11]. Therefore, it is essential to explore the effect of meta-awareness on MW. Moreover, previous studies on the account of meta-awareness distinguish two aspects of meta-awareness (e.g., meta-awareness knowledge and meta-awareness monitoring). Meta-awareness knowledge is the knowledge of general facts about how the brain works; meta-awareness monitoring is to track one’s mental operation; meta-awareness control is to use the results of meta-awareness monitoring to modulate performance [8,12,13].

Many previous studies used typical sustained attention response tasks (typical SART) to explore MW. SART is similar to the go/no-go task. A typical SART stimulation or experimental task is a digit from 0 to 9. Participants are required to press a button every time a digit except “3” appears on a screen. When digit “3” appears on the screen, participants have to refrain from responding. During the task, participants are asked with a random probe what they are experiencing and whether they are aware of their minds having wandered [14,15,16]. Typical SART captures MW events using probes. During the process of MW captured by probes, participants failed to notice MW, their attention was still focused on perception, and there was no explicit idea of the current contents of their consciousness. Such MW events obtained through external clues do not involve meta-awareness. Therefore, when examining MW, most ERP studies using typical SART only focus on the differences between MW and non-MW, and ignore the influence of meta-awareness on MW. However, Schooler et al. claimed that meta-awareness plays an important role in MW. It may directly contribute to the regulation of MW because the mind is intermittently aware of MW [11]. However, MW may be temporary and difficult to categorize [17]. Kawagoe and Kase asked participants to report their psychological experiences, and their answers were categorized post hoc. They found many task-related thoughts or task-related psychological experiences during the typical SART. Additionally, they used a data-driven approach to further analyze the experiences based on the participants’ answers and found that meta-awareness ability possibly contributed to MW [18]. As far as we know, there is only one functional Magnetic Resonance Imaging (fMRI) study that has examined the relationship between meta-awareness and MW by typical SART. Christoff et al. used two probes and asked participants whether their attention was focused on the task and whether they knew where their attention was focused. They found that MW with meta-awareness activated brain regions similar to those observed during MW without meta-awareness (executive network: dorsal anterior cingulate cortex and lateral prefrontal cortex; default network: ventral anterior cingulate cortex, posterior cingulate/precuneus, and temporoparietal cortex). But MW with meta-awareness was associated with weaker activation in the two networks [16]. Therefore, MW with and without meta-awareness had similar activation patterns in fMRI studies during the typical SART. Furthermore, during periods of MW in the reading task, the control network activity showed a significant increase in activity in the right inferior frontal junction, the right dorsal pre-motor cortex, and the bilateral anterior cingulate cortex. The study suggested that the control network associated with MW was somewhat right-lateralized. Moss and colleagues believed that the reading task involved minimum participation in the control process. These processes are mainly left-lateralized due to language specialization, which will be one of the reasons for right lateralization [19].

Compared with fMRI, event-related potentials (ERPs) have a higher temporal resolution and are another method commonly used to study the neural mechanism underlying MW. Previous ERP investigations have shown that when participants were MW during a typical SART period, the perceptual processes of external stimuli were reduced. When participants performed a typical SART, the amplitudes of visual P1 and auditory N1 decreased during MW [14,15]. In addition, when participants were not MW, the P1 amplitudes of the right hemisphere were greater than those of the left hemisphere. When participants were MW, the N1 amplitudes in the left hemisphere decreased [20]. However, the positive component (P2) at approximately 200 ms after the presentation of the stimulus was significantly higher during MW. This result suggested attention disengagement is related to the stimulation process and low alertness during MW [7]. Thus, the changes in earlier components of ERPs’ amplitudes seem to be related to the participants’ attention, indicating that the participants’ attention had drifted away from the task at hand. 

The amplitudes of later components of ERPs also change during the MW period. A study reported that when participants were MW during typical SART, the amplitudes of the P300 decreased [21], indicating a decrease in attentional resources for stimulus processing. P300 is a large-amplitude positive ERP component that peaks at 300–600 ms following the presentation of stimuli [22,23]. The amplitude of P300 is related to the intensity of processing [24], which may increase with task difficulty and effort [25]. Previous ERP studies have shown that N2 is a negative potential that appears about 200–350 ms after the start of stimulation, and is related to executive function [26,27,28,29,30,31]. Frontal N2 is particularly related to the anterior cingulate cortex activation in the prefrontal cortex [32]. The anterior cingulate cortex may be a crucial region for cognitive control or conflict. Previous studies have shown that N2 amplitudes on the frontal electrodes were greater than those of the central electrodes [30,33]. Moreover, N300 might be similar to N2 [34], which is a critical indicator in conflict monitoring [34,35]. Thus, N300 is also related to executive function.

We aim to explore the neural markers of MW from the temporal perspective. In order to capture the dynamic of the thinking process during the task, we modified the typical SART based on a dynamic framework used to understand mind wandering [36]. Specifically, we superimposed dynamic responses during a typical SART, and identified different kinds of MW based on whether or not the participant was aware of their MW. We required participants to press a button whenever they realized that they were MW during the task. This was classified as self-caught MW, and the MW identified by probes was defined as probe-caught MW. During modified SART (mSART), participants can voluntarily report dynamic changes in their attention.

Further, we recorded ERPs during mSART. In previous ERP studies [7,14,15,21], all the components reported occurred within a time window of 100–450 ms. Therefore, we focused on the changes in relevant cognitive ERP components within this specific time window, including P1 or N1, P2 or N2, and P300 or N300. Furthermore, since these components were based on the typical SART, these components should be an integrated representation of self-caught MW and probe-caught MW. However, under the mSART, when self-caught MW and probe-caught MW are separated, the situation would be different. For this reason, we hypothesized that due to the involvement of meta-awareness during mSART, the ERPs within the time window will be separated under the conditions of self-caught MW and probe-caught MW. Therefore, there will be a different pattern observable for self-caught and probe-caught MW.

## 2. Materials and Methods

### 2.1. Participants

A total of 40 volunteers from Southwest University in China (27 females, 13 males; age 18–25 years old, M = 21.36 years, and SD = 2.20) signed written consent forms before participating in the experiment. Participants were asked to avoid any substances or medications that might potentially affect their concentration. Only participants with no history of major psychological disorders were included in the study. All participants were right-handed, with normal or corrected-to-normal vision. Before giving written consent to participate, all participants were asked to read the instructions and were allowed to ask questions about the experiment. The study was approved by the Ethics Committee of Southwest University.

### 2.2. Procedure

**Figure 1:**
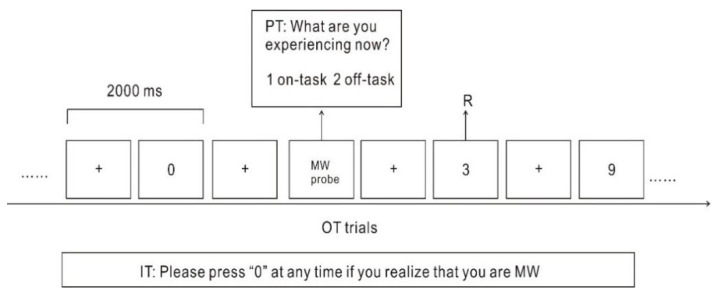
The modified sustained attention to response task (mSART). OT: on-going task. PT: passive task. IT: introspective task. Participants were required to press button “1” every time a digit except “3” appeared on the screen. Participants were asked to press the button “0” as soon as they realized they were MW (self-caught MW). When the probe appeared on the screen, if participants selected “off-task”, this indicated that they were MW (probe-caught MW).

Participants completed tasks in a quiet room specially designed for electroencephalography (EEG) experiments. The E-prime-based version of the mSART was used. The numbers from “0” to “9” appeared in the center of the screen in a random order, with a digit presented every 2 s. Participants were required to press a button labelled “1” every time any digit except “3” appeared on a screen. When the digit “3” appeared on the screen, participants had to refrain from responding. Targets consisting of the digit “3” constituted 5% of the all digits that appeared during the test. The digit “3” appeared a total of 64 times in the course of the test [37]. During the task, participants were asked to press a button labelled “0” as soon as they realized they were MW (Figure 1). The MW captured by pressing “0” was classified as self-caught MW. Occasionally, probes asked participants “What are you experiencing now?” (1. on-task; 2. off-task). If participants selected “off-task”, which indicated that they were MW, then it was classified as probe-caught MW. The total number of probes was 64, and the content of each probe was the same. The task lasted approximately 65 min and was divided into four blocks of 14 min each. At the end of each block, participants were permitted to rest for 3 min and move around.

The stimulus was presented on a 19-inch Dell computer monitor with the center of the screen set at eye level. Participants were instructed to remain as still as possible and minimize blinking to reduce experimental artifacts during EEG data collection.

### 2.3. ERP Recording and Analysis

ERP data were recorded from a 64-electrode cap positioned according to the 10–20 system for electrode placement, with the linked reference on the left and right mastoids, and a ground electrode (Brain Products, Gilching, Germany) placed on the medial frontal surface. The horizontal electrooculogram (HEOG) was recorded by placing the electrodes outside both eyes, and the vertical electrooculogram (VEOG) was recorded by placing the electrodes up and down on the left eye. The impedance of each electrode remained below 5 kΩ.

Data processing was performed with MATLAB R2014a using the EEGLAB toolbox14.1.1b [38]. Data were processed offline after the continuous recording of ERP. Based on a previous study [28], we first down-sampled the data from 500 Hz to 256 Hz and performed high-pass filtering at 0.01 Hz and low-pass filtering at 45 Hz. We selected the left and right mastoid as the re-reference. Data were epoched from 0.2 s before the start of the stimulation to 2 s after the presentation of the MW (for both self-caught MW and probe-caught MW), and baseline correction was performed according to the pre-stimulation interval. Eye movement artifacts (blinks and eye movements) were rejected offline. Trials with electrooculographic (EOG) artifacts (ocular movements and blinking), as well as artifacts due to amplifier clippings, bursts of electromyography activity, or peak-to-peak deflections exceeding ±80 μV were excluded from the average. Components including EOG artifacts and head movements were removed from the independent component analysis (ICA) results after visual inspection.

The self-caught MW epochs (recorded when button “0” was pressed) were compared to the epochs that were acquired from the response to all digits when not MW (i.e., when button “1” was pressed). We took epochs during which participants reported they were not MW (i.e., selected “1. on-task” when probes appeared on the screen) as a baseline against which to compare to probe-caught MW (selected “2. off-task” when probes appeared on the screen). According to the topographic distribution of the grand-averaged ERPs’ activities, the ERPs and their time windows were as follows: N1 (100–160 ms), P2 (180–230 ms), N300 (270–350 ms), and P300 (350–450 ms). The following electrode positions were selected: frontal (F3, F4), frontal-central (FC3, FC4), central (C3, C4), central-parietal (CP3, CP4), parietal (P3, P4), and parietal-occipital (PO3, PO4). Four repeated-measure ANOVAs—for the 2 attentional states (MW and non-MW), 2 lateralities (left and right), 2 causalities (anterior and posterior) and 3 electrode sites (left anterior: F3, FC3, C3; left posterior: CP3, P3, PO3; right anterior: F4, FC4, C3; right posterior: CP4, P4, PO4)—were conducted on the mean amplitudes of N1, P2, N300, and P300. All analyses were conducted using SPSS20.0. The *p*-values were computed for deviations in all analyses based on the Greenhouse–Geisser method. Post-hoc t-tests with Bonferroni adjustments were conducted for multiple comparisons. We conducted outlier analyses on EEG data using ±3 SDs and 3 participants were excluded from the final data analysis.

## 3. Results

### 3.1. Behavioral Results

The mean accuracy of digits other than “3” (NACC) was 0.95, and the mean accuracy of digit “3” (TACC) was 0.54. The paired sample t-test showed NACC was significantly greater than TACC, *t* (36) = 13.08, *p* < 0.001, Cohen’s d = 4.30. The reaction times (RTs) in self-caught MW were significantly greater than those in non-self-caught MW, *t* (36) = 8.46, *p* < 0.001, Cohen’s d = 2.78. However, there was no significant difference in RTs between probe-caught MW (1913.16 ms) and non-probe-caught MW (1713.30 ms), *p* = 0.12.

In addition, we investigated the relationship between task accuracy and MW at the individual level. The results showed that NACC was negatively correlated to self-caught MW, r = −0.67, *p* < 0.001. TACC was negatively related to self-caught MW (r = −0.47, *p* = 0.004) and probe-caught MW (r = −0.36, *p* = 0.03).

### 3.2. ERP Results

**Figure 2:**
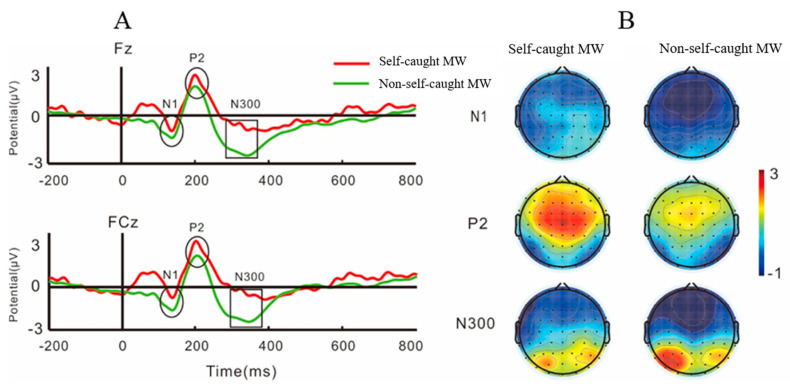
The mean amplitudes of N1, P2 and N300 (**A**) and topography plots (**B**) during self-caught MW and non-self-caught MW.

**Figure 3:**
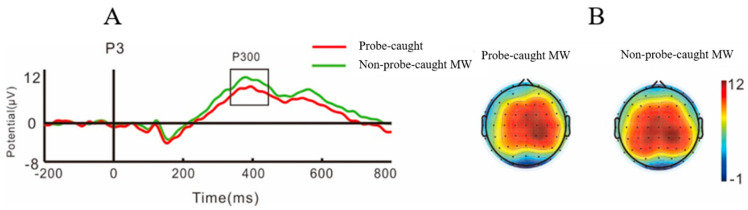
The mean amplitudes of P300 (**A**) and topography plots (**B**) during probe-caught MW and non-probe-caught MW.

The grand average ERPs of N1, P2, and N300 and the topography plots during self-caught MW and non-self-caught MW are shown in Figure 2. The mean amplitudes of P300 and the topography plots during probe-caught MW and non-probe-caught MW are shown in Figure 3.

#### 3.2.1. Self-Caught MW

Results for N1 in a window of 100–160 ms showed an interaction of attentional state (self-caught MW and non-self-caught MW) and caudality and electrode sites, *F* (1, 36) = 9.47, *p* = 0.001, *η^2^* = 0.21. For a significant attentional state by caudality interaction, *F* (1, 36) = 5.61, *p* = 0.02, *η^2^* = 0.14, follow-up simple effect analysis found the mean amplitudes of N1 were significantly reduced during self-caught MW compared to non-self-caught MW at both anterior and posterior regions, all *p* < 0.05. In addition, anterior regions elicited greater N1 amplitudes, *p* = 0.04. We did not find any interaction between attentional state and electrode sites [*F* (1, 36) = 0.69, *p* = 0.44, *η^2^* = 0.01] or any interaction between caudality and electrode sites [*F* (1, 36) = 0.17, *p* = 0.71, *η^2^* = 0.005]. Results showed an interaction between attentional state and laterality and electrode sites, *F* (1, 36) = 5.02, *p* = 0.01, *η^2^* = 0.12. We did not find any interaction between attentional state and laterality [*F* (1, 36) = 0.33, *p* = 0.57, *η^2^* = 0.009], between attentional state and electrode sites [*F* (1, 36) = 0.69, *p* = 0.44, *η^2^* = 0.02], or between laterality and electrode sites [*F* (1, 36) = 0.33, *p* = 0.65, *η^2^* = 0.009]. Results also showed an interaction between attentional state and laterality and caudality, *F* (1, 36) = 6.28, *p* = 0.02, *η^2^* = 0.15. With regard to the interaction between laterality and caudality, *F* (1, 36) = 7.87, *p* = 0.01, *η^2^* = 0.18, the follow-up simple effect analysis showed that N1 amplitudes on right anterior regions were greater than those on the right posterior regions, *p* = 0.03. In addition, the N1 amplitudes on the left posterior regions were greater than those on the right posterior regions, *p* = 0.001.

Results for P2 in a window of 180–230 ms revealed an interaction between attentional state and caudality and electrode sites, *F* (1, 36) = 15.44, *p* < 0.01, *η^2^* = 0.3. For the interaction between caudality and electrode sites, *F* (1, 36) = 7.44, *p* < 0.01, *η^2^* = 0.17, the follow-up simple effect analysis revealed P2 amplitudes on the anterior regions were significantly greater than those on the posterior regions, all *ps* < 0.05. We did not find any interaction between attentional state and caudality [*F* (1, 36) = 2.75, *p* = 0.11, *η^2^* = 0.07] or any interaction between attentional state and electrode sites [*F* (1, 36) = 1.36, *p* = 0.26 *η^2^* = 0.04]. Results also showed a marginal interaction between attentional state and laterality and caudality, *F* (1, 36) = 3.85, *p* = 0.057, *η^2^* = 0.1. With regard to the interaction between attentional state and laterality, *F* (1, 36) = 6.07, *p* = 0.02, *η^2^* = 0.14), the follow-up simple effect analysis showed that P2 amplitudes during self-caught MW were greater than those during non-self-caught MW, all *p* < 0.05. We did not find any interaction between laterality and electrode sites or interaction between attentional state and laterality and electrode sites, all *p*s > 0.05. We did not find any interaction between laterality and caudality, *F* (1, 36) = 0.66, *p* = 0.42 *η^2^* = 0.02.

Results for N300 in a window of 270–350 ms showed an interaction between attentional state and laterality and caudality, *F* (1, 36) = 10.71, *p* = 0.002, *η^2^* = 0.23. For the interaction between attentional state and caudality, *F* (1, 36) = 14.85, *p* < 0.01, *η^2^* = 0.29, follow-up simple effect analysis found N300 amplitudes on anterior regions were greater than those on the posterior regions during self-caught MW and non-self-caught MW, all *ps* < 0.01. In addition, compared to non-self-caught MW, N300 amplitudes during self-caught MW were lower on the posterior regions, *p* = 0.03. For the interaction between laterality and caudality, *F* (1, 36) = 5.51, *p* = 0.02, *η^2^* = 0.13, the follow-up simple effect analysis showed that N300 amplitudes on the anterior regions were greater than those on the posterior, all *p* < 0.01. However, there was no significant difference between the left and right hemispheres, all *p* > 0.05. We did not find any interaction between attentional state and laterality and electrode sites [*F* (1, 36) = 1.14, *p* = 0.31, *η^2^* = 0.03], or any interaction between attentional state and caudality and electrode sites [*F* (1, 36) = 1.83, *p* = 0.18, *η^2^* = 0.05].

#### 3.2.2. Probe-Caught MW

The results for P300 in the 350–450 ms window showed an interaction between attention state and laterality, *F* (1, 36) = 5.94, *p* = 0.02, *η^2^* = 0.14, and the subsequent simple effect analysis showed that the P300 amplitudes during probe-caught MW were marginally lower on the left hemisphere compared to those during non-probe-caught MW, *p* = 0.06, and P300 amplitudes during probe-caught MW in the right hemisphere were greater than those in the left hemisphere, *p* = 0.05. We did not find any interaction between attention state and causality [*F* (1, 36) = 2.93, *p* = 0.1, *η^2^* = 0.07], or any other interactions, all *p* > 0.05.

## 4. Discussion

The current study aimed to use the mSART to characterize the neural markers of self-caught MW and probe-caught MW. Participants were asked to respond to a series of stimuli, and to press a certain button when they realized their minds were wandering. During the task, we collected all instances of MW caught as a result of participants’ meta-awareness (self-caught MW) and probes (probe-caught MW). The RTs in self-caught MW were significantly greater than those in non-self-caught MW. In addition, NACC was significantly correlated to self-caught MW, and TACC was related to both self-caught MW and probe-caught MW during the mSART. The ERP results showed that N1 and N300 amplitudes decreased during self-caught MW, while P2 amplitudes increased; but P300 amplitudes decreased during probe-caught MW. Moreover, N1, P2, and N300 amplitudes in anterior regions were greater than those in posterior regions. P300 amplitudes during probe-caught MW in the right hemisphere were greater than those in the left hemisphere. These findings suggested that the neural markers of self-caught MW and probe-caught MW are different. The change in ERPs occurred approximately 100–350 ms after the start of stimulus between self-caught MW and non-self-caught MW. In the same time window, there was no difference between the ERPs of probe-caught MW and the non-probe-caught MW. The neural markers of probe-caught MW were different from those of non-probe-caught MW after 350 ms.

Based on these findings, we believe that there may be two reasons for the different patterns generated by self-caught and probe-caught MW. First, self-caught mind-wandering was measured by participants’ awareness when the stimulus (number 0–9) appeared in the center of the screen, while probe-caught mind-wandering was measured by probes (in the form of a question). Therefore, it is possible that different visual stimuli may induce different patterns. Second, and more importantly, measuring self-caught MW involves the participation of the participants’ meta-awareness, while probe-caught MW is only identified passively. So meta-awareness may have different effects on self-caught MW and probe-caught MW neural patterns. We believed that meta-awareness might play a critical role during mSART in the current study. We discuss this below.

A previous study showed that individuals who display less MW might hold more meta-awareness beliefs and judgments, and a demonstrate greater tendency toward meta-awareness monitoring [39]. In addition, less MW was related to less dysfunctional meta-awareness [39,40]. Therefore, the fact that NACC was only negatively related to self-caught MW might be due to the involvement of meta-awareness. SART is similar to the go/no-go task. The task required participants to inhibit their response when the digit “3” appeared on the screen, which involved their inhibition ability. Therefore, the finding that TACC was negatively related to both self-caught MW and probe-caught MW might be explained by the view that MW represents a failure of executive control [41].

N1 reflects the facilitation of task-relevant processing [42]. A previous study found that the amplitudes of N1 elicited by auditory stimuli decrease when selective attention is oriented away from the task at hand [43]. Moreover, N1 has been related to a stimulus discrimination process [44,45]. In the current study, we found that N1 mean amplitudes were reduced during self-caught MW, which suggested a disengagement of attention from stimuli processing and an attenuation of attention early in the 100–160 ms time window after stimulus presentation during self-caught MW. The finding might support the view that MW consumes cognitive resources, drawing them away from one’s primary task in the absence of effective meta-awareness monitoring [1]. In addition, we found that the amplitudes of P2 were greater during self-caught MW compared to non-self-caught MW, which was consistent with previous studies. For example, a study by Braboszcz and Delorme used a breath-counting task and instances of MW were recorded by means of participants’ introspection. They found that amplitudes of P2 during MW were larger than non-self-caught MW, indicating an attentional disengagement toward stimuli processing and decreased alertness during MW [7]. Similarly, Naatanen et al. reported that an increase in the P2 component was related to the disengagement of participants’ attention toward stimuli [46]. In addition, an increase in the P2 component was characteristic of the sleep onset period [47]. Crowley and Colrain showed that when the level of participants’ attentiveness increased, the amplitudes of P2 decreased [48].

It has been demonstrated that MW is related to meta-awareness. One study found that the higher the frequency of MW, the lower the cognitive confidence (measured by a metacognitions questionnaire) [40], which is consistent with the theory that MW may be caused by the failure of executive control [41]. Individuals who experience more MW due to failures to perform executive control might only become aware of that they are MW some time after it begins, which might result in the MW being judged to have occurred unintentionally [40]. Interestingly, we found that the amplitudes of N300 decreased during self-caught MW. The results showed that self-caught MW not only reduces attention resources but also that cognitive control might be affected by self-caught MW. Some researchers believe that the composition of N300-400 may be similar to that of N2, and the dipole source analysis of N300-400 is limited to the anterior cingulate cortex [34]. The anterior cingulate cortex might be a key region for cognitive control or conflict. Since N300 is a critical index in conflict monitoring [34,35], it can be used for the detection of interference stimuli in the early stage of cognitive processing [49]. Previous studies have shown that meta-awareness can track one’s mental operations and modulate performance [8,13]. Therefore, our findings on N300 might indicate that, due to the involvement of meta-awareness, self-caught MW can affect cognitive control and conflict monitoring while carrying out a task. Thus, our research results also support the view that MW represents the failure of execution, which is determined by the existence of automatically generated thoughts in response to the environment and mental cues, and the ability of the executive-control system to deal with such disturbances [41].

ERPs within the time window of 100–450 ms were separated according to whether they were self-caught MW or probe-caught MW. We did not find a difference between self-caught MW and non-self-caught MW after 350 ms, but we found that probe-caught MW was different from non-probe-caught MW during this time window. Compared with non-MW, the mean amplitudes of P300 during probe-caught MW decreased in the 350–450 ms window. Previous studies have also found a decrease in P300 amplitudes during MW in migraineurs [14]. P300 amplitudes were considered to be more closely related to processing intensity [24], and might increase with the difficulty and effort of the task [25]. A larger P300 amplitude suggests that more attention resources are being allocated to the current task [27,28,29,30,50]. Therefore, P300 may be related to complex cognitive processes. However, detecting probe-captured MW may involve another complex cognitive process, such as recall processing. In our study, when the probes appeared on the screen, participants might have recalled their prior attentional state before the current probe. Therefore, the difference in P300 may be related to the high-level processing difference between probe-caught MW and non-probe-caught MW.

The MW-related effects of early ERPs, N1, and P2 were consistent with the process of perceptual decoupling as a general accompaniment of MW. Our findings were consistent with previous studies [15,20,51]. The changes in N1 and P2 may suggest functional separation, which may have important implications for attention patterns in cognitive neuroscience. When participants became aware that they were MW, they realized that there were conflicts between on-task and off-task thoughts during the current task. Therefore, they needed to bring their attention back to the current task based on their meta-awareness monitoring. According to current research, N300 might be related to the meta-awareness of conflict resolution. In particular, the relevant results from our analysis showed that NACC was negatively related to self-caught MW, indicating that the mental shifts between on-task and off-task thoughts might impact task performance, resulting in more mental shifts and lower task accuracy. In summary, we believe that meta-awareness may capture the dynamic changes of attention during self-caught MW and plays the role of conflict monitoring.

Alpha power is commonly used to study neural markers of mind-wandering. For example, Jin et al. used a machine-learning algorithm to explore the predictive factors of mind-wandering. They found alpha power was the most reliable index for predicting MW [52]. Compton et al. found that alpha power during MW was significantly higher compared levels during non-MW periods. They suggested that alpha power could be a valuable tool for studying momentary fluctuations in MW [53]. On the other hand, Braboszcz and Delorme found that alpha power decreased during mind wandering [7]. In the current study, we did not find a difference in alpha power between MW and non-MW states (details can be found in Supplements), which is consistent with the previous study [20]. Although the critical variables contributing to the various findings in previous studies have not yet been fully determined [53], we believe that the types of stimuli and tasks may be important factors.

Several limitations of our study should be acknowledged. First of all, we used ERPs solely to study MW, whereas previous studies of MW have always been single-modal studies. Multi-modal neuroimaging techniques should be applied to study MW in future studies. For example, simultaneous EEG–fMRI has the advantages of both EEG and fMRI. Secondly, instances of MW are usually identified through self-reporting or probes. There is no consolidated and objective indicator of MW. Therefore, objective criteria for defining MW should be established in the future. For example, real-time EEG or fMRI readings could be used to distinguish whether or not participants were MW. Third, the stimuli used in this study were digits, but we also used digits as the response keys, which may have affect the participants’ performance in the task. Future studies should use different types of symbol for the stimulus and the response keys. Fourth, the current study did not distinguish probe-caught MW with and without meta-awareness. Although participants did not report MW in the three trials before the probe-caught MW in the current study, it is necessary to further distinguish probe-caught MW with and without meta-awareness in future studies.

## 5. Conclusions

We conclude that MW corresponds to a state of low vigilance and reduced attention. The difference in neural markers between self-caught and non-self-caught MW may occur in the early ERP component. However, the difference of neural markers between probe-caught and non-probe-caught MW may occur in a later ERP component. As far as we know, this is the first study investigating how ERP correlates with self-caught MW and probe-caught MW. As such, it provides new insights into the association between MW and neural markers and extends the previous literature by identifying variations in neural markers between the two types of MW.

## Data Availability

The data presented in this study are available on request from the corresponding author.

## Supplementary Materials

The following are available online at https://www.mdpi.com/article/10.3390/brainsci11101329/s1, Figure S1: TF results at FCz.
