# Supplementary material for: The Neural Markers of Self-Caught and Probe-Caught Mind Wandering: An ERP Study
Source: Brain Sci. 2021 Oct 8;11(10):1329. doi: 10.3390/brainsci11101329 (PMC8534158; doi:10.3390/brainsci11101329)
Supplement: Supplementary file 1 [file brainsci-11-01329-s001.zip › brainsci-1387724-supplementary.pdf]

The neural markers of self-caught and probe-caught mind wandering: an ERP study

Yong Liu [1,2], Jia Zhao [1,2], Xinqi Zhou [3], Xiaolin Liu [1,2,4], Hong Chen [1,2] and Hong Yuan [1,2,*]

[1] *Key Laboratory of Cognition and Personality (Ministry of Education), Southwest University, Chongqing, 400715, China; liuy0768@swu.edu.cn (Y.L.);* jiazhao@swu.edu.cn (J.Z.); linzi306093@email.swu.edu.cn (X.L.); chenhg@swu.edu.cn (H.C.)

[2] *School of Psychology, Southwest University, Chongqing, 400715, China*

[3] School of Life Science and Technology, *University of Electronic Science and Technology of China;* Chengdu, 611731, China, 201711090103@std.uestc.edu.cn

[4] Chongqing *Institute of Foreign Studies, Chongqing, 400715, China*

* Correspondence: yuanyh@swu.edu.cn (H.Y.)

Alpha power analysis

Short-time fourier transform (STFT) is applied to calculate the time-frequency results, where the window length is 0.25 s (sampling rate is 256 Hz) is used during the calculation. As the band-pass filter is set as 0.1 – 30 Hz during the preprocessing, the frequency range for the time-frequency analysis is set as 1 – 30 Hz. The relative variation (RV) of power is employed for the baseline-correction of the TF results, which has the form as $RV = \left(P_i - \overline{P}\right)/\overline{P}$. In the fomula, Pi is the power of each point in the time-frequency domain, and $\overline{P}$ is the average power of baseline. In this study, 200 ms before the stimuli is selected as the baseline, which is the same as in the ERP analysis. In addition, the differences between the power of mind wandering and non-mind wandering is compared. As the statistics results are not significant for their differences, only the subtraction results of TF analysis (mind wandering - non-mind wandering) are shown in figure 1 below. From the Fig 1, it can be found that the alpha power of mind wandering tends to be stronger than that of the non-mind wandering almost at the whole time sequence at FCz.

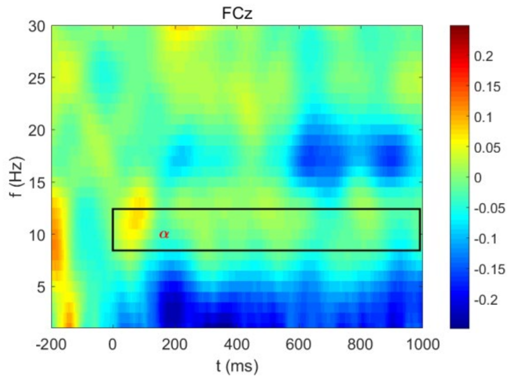

Figure S1 TF results at FCz
